# Commemorating the 40-Year Journey of the School of Medical Sciences, Universiti Sains Malaysia

**DOI:** 10.21315/mjms2019.26.2.1

**Published:** 2019-04-30

**Authors:** Shaiful Bahari Ismail, Rosline Hassan, Kamarul Aryffin Baharuddin, Abdul Razak Sulaiman, Kamaruddin Jaalam, Wan Hazabbah Wan Hitam, Wan Zalawati Mohd Noor, Wan Nur Fajrina Wan Azmi, Noraida Yusoff, Muzaimi Mustapha

**Affiliations:** School of Medical Sciences, Universiti Sains Malaysia, USM Health Campus, Kubang Kerian, Kelantan, Malaysia

**Keywords:** School of Medical Sciences, USM, 40-year anniversary, compassionate professionals

## Abstract

The School of Medical Sciences of Universiti Sains Malaysia (USM) is the launching pad for this journal. From the school’s humble beginning at the USM Main Campus in Pulau Pinang, Malaysia, it has grown in stature at its current location in the USM Health Campus, Kubang Kerian, Kelantan, Malaysia. Commemorating its 40th anniversary, this editorial aims to recollect, although not exhaustively, the wealth of returns for the USM, as well as for the nation, which the school has managed to deliver in that period. Resolute to its vision and mission, this article highlights the outstanding accomplishments in various core aspects of the school’s academic, research and professional growth as we continually strive to train globally competitive and compassionate medical graduates, medical specialists and scientists, skilled to serve nation’s needs and broader markets worldwide. Currently guided by the Malaysian Higher Education Blueprint (2015–2025), the school shall remain ingenious in its duties in the many more years to come, as we head for a world-class trajectory.

## Introduction

Since its inception in 1979, the School of Medical Sciences of the Universiti Sains Malaysia (USM) was found in a temporary makeshift building in the USM Main Campus in Pulau Pinang, Malaysia. Being the third medical school in Malaysia at the time, it has been relocated three times within the USM Main Campus before finally settling in 1990 in a brand new USM Health Campus at its current and permanent location in Kubang Kerian, Kelantan, Malaysia (1). Starting as a lone academic establishment along with the USM teaching hospital, it served as the launching pad to establish other academic schools, namely the School of Dental Sciences, School of Health Sciences, Institute for Molecular Medicine (INFORMM) and, pertinent so, the launch of this journal (2–3).

This special editorial aims to highlight key accomplishments of this medical school on its 40th anniversary, although by no means exhaustive, with remarkable milestones (Table 1). We strive to continually provide a globally-recognised and locally-effective medical education and health care in the region that adheres to the highest standards and to lead translational research for a sustainable ecosystem.

## 40 Years of Undergraduate Studies

The only undergraduate programme offered by the school is the Doctor of Medicine (MD) programme—a 5-year course designed to be integrated, problem-based and community-oriented. Since its inception until 2014, the programme was categorised into three phases, which were linked by the concept of ‘spiralling’. The first spiral involved most of the learning and activities in phase 1, which converged and reinforced upon the second phase as the second spiral and so on. This ‘spiral’ concept enabled the school to implement the philosophy of both horizontal and vertical integration of subjects/disciplines.

In 2014, the curriculum underwent revision whilst maintaining the spiral concept. The phases were categorised into two phases only— years 1–2 in phase 1 and years 3–5 in phase 2. The new curriculum uses a credit unit system for the entire programme, which is the first of its kind in the country. The total credit unit for the core courses is 174. To enhance work preparedness amongst graduates, an intern-shadowing programme—Shadow House Officer Training Schedule (SHOTS)—was introduced in 2018. SHOTS serves as a training ground for final-year students to augment essential skills and the professional role as an intern under the guidance of educators. With the motto ‘Compassionate Professional’, the school pledges to nurture holistic graduates. The Personal and Professional Development Programme is another unique programme under the School of Medical Sciences to equip students with soft skills and humanity.

Being the first school in the nation to adopt an innovative curriculum, the school is known for its Community and Family Case Studies programme. Since its inception in 1980, the research-based community programme has promoted the students’ community participation through multiple health-promotion projects. The community projects are intertwined throughout the curriculum, enabling students to engage and empower the community by guiding them to proactively act on common diseases such as diabetes, tuberculosis and dengue.

Moreover, the school has registered a historic achievement by launching an offshore medical programme in Belgaum, India, since 2010; this joint-venture programme is run in collaboration with Karnataka Lingayat Education (KLE) and was named as USM-KLE International Medical Program (USM-KLE IMP). In addition, the School of Medical Sciences has opened the programme to international students since 2016. Currently, 13 international students are studying medicine in the school from various countries. Guided by the vision of ‘*Medicine for Sustainable Tomorrow*’, the school is optimistic in producing competent and safe doctors for the country.

## 40 Years of Postgraduate Studies

The coordination of all postgraduate studies is led by the Deputy Dean of Postgraduate Studies with the school’s dedicated Postgraduate Office that works closely with the Institute of Postgraduate Studies. The school offers a broad range of postgraduate programmes on medical and health-related disciplines. Three different modes of postgraduate studies are the research-mode, coursework-mode and mixed-mode. Whilst targeting local students, the school also attracts up to 25% of foreign students (the majority for doctorate levels in research-mode). The Integrated Neuroscience Programme (INP), which is a mixed-mode programme, is one of the popular programmes, with 30.8% international students’ enrolment.

The coursework-mode (clinical specialists’ programme) is the core programme of the school, representing 79% of its postgraduate students for 22 programmes with the capability to train approximately 800 candidates at a time. The current trainers-to-trainee ratio is 1:2.21. These programmes comprise Masters of Medicine (M-Med), Master of Surgery (MS), Master of Pathology and Master of Public Health (MPH)/Doctor of Public Health (DrPH). The training involves 4–7 year structured programme and typically caters to medical officers who are also serving their clinical service for hospitals where they are posted. The Department of Medicine initiated the first programme known as the Master of Medicine (Internal Medicine) in 1988, and the first batch graduated in 2002. Since then, many other disciplines have been rolled out. Briefly, the milestones for various medical specialists’ programmes are as follows: 1991—paediatrics, surgery, orthopaedics and obstetrics & gynaecology; 1992—pathology with anatomic pathology, haematology, medical microbiology and chemical pathology; 1993—anaesthesiology; 1995—radiology and family medicine; 1996—psychiatry; 1997— ophthalmology and otorhinolaryngology; 1998— community medicine, emergency medicine and master of pathology (immunology); 2001— neurosurgery, reconstructive surgery; 2009— DrPH and 2010—pathology (clinical genetics).

With a rising demand for specialists in the country, and within the backdrop of the scarcity of medical officers operating public hospitals (4), formalised win–win collaboration with the Ministry of Health (MOH) was sealed in 1996. Students can opt (or the choice made for them) for ‘open’ or ‘close’ system of training. In the ‘open’ system, students spend 2 years in MOH hospitals under co-supervision of MOH specialists (honourary lecturers) and the next 2 years in the university supervised by the USM lecturers. In the ‘close’ system, students spend the entire training period in the university hospital. USM started enrolling foreign candidates for the clinical specialists programmes in 2000. Eventually, these specialist training programmes obtained recognition from several countries. By December 2018, USM has produced 2435 clinical specialists in various fields, including 71 specialists from countries like Saudi Arabia, UAE, Iran, Iraq, Yemen, Nigeria, Sudan, Palestine, Maldives, Mauritius, Fiji, Indonesia and India.

In addition, the research-mode programme witnessed its first two Doctor of Philosophy (PhD) candidates in microbiology and immunology graduating in 1993; since then, this number has increased manifold over the years. Up to December 2018, the School has produced 248 Master of Science (MSc) and 150 PhD graduates. As a commitment for quality assurance, the school’s diagnostic and research laboratories are accredited with MS ISO 9001:2008, MS ISO 15189 and MS ISO 17125, respectively. Finally, the mixed- and coursework-mode (non-clinical speciality) programmes are being conducted throughout the course and relevant research work. Of note, MSc (Sports Science) was the first mixed-mode programme offered in 1997. Other programmes comprise MSc (Clinical Anatomy), MSc (Medical Statistics) and MSc (Medical Education). The latter two programmes are not offered elsewhere in the country. In 2012, the school started offering INP, the first of its kind in the country for fundamental neuroscience postgraduate education, leading to the award of Master of Neuroscience (MNeurosc) and Doctor of Neuroscience (NeuroscD). Master of Cognitive Neurosciences and Integrated Master of Psychology (Clinical) and Doctorate Psychology (Clinical Psychology and Clinical Neuropsychology; joint degree with Universiti Pendidikan Sultan Idris) are the most recent programmes which started in September 2018.

## 40 Years of Research and Publications

When the school was in the USM Main Campus, the data on research records were scant. The primary focus was on teaching and learning, compared with research. When the school moved to Kelantan, the first compilation of data was available for 1990–2000, during which the total amount of grant acquisition was RM21,916,878. The names of prominent earlier researchers of the school were mainly led from the Department of Microbiology; their names are worth mentioning specifically as they laid the foundation of research and enrolled the schools’ first few PhD students. Although they no longer served the school, their legacy remains. Before 2000, the Research and Development (R&D) unit was under the school’s administration office with a scope of managing application and human research ethics; since then, the school’s R&D unit has become an entity in itself under the direction of Deputy Dean Research. By 2004, a research platform was set up for the Health Campus, which entirely managed the human research ethics affair, followed by the official animal research ethics Institutional Animal Care and Use Committee (IACUC) setup in 2018.

**Figure 1:**
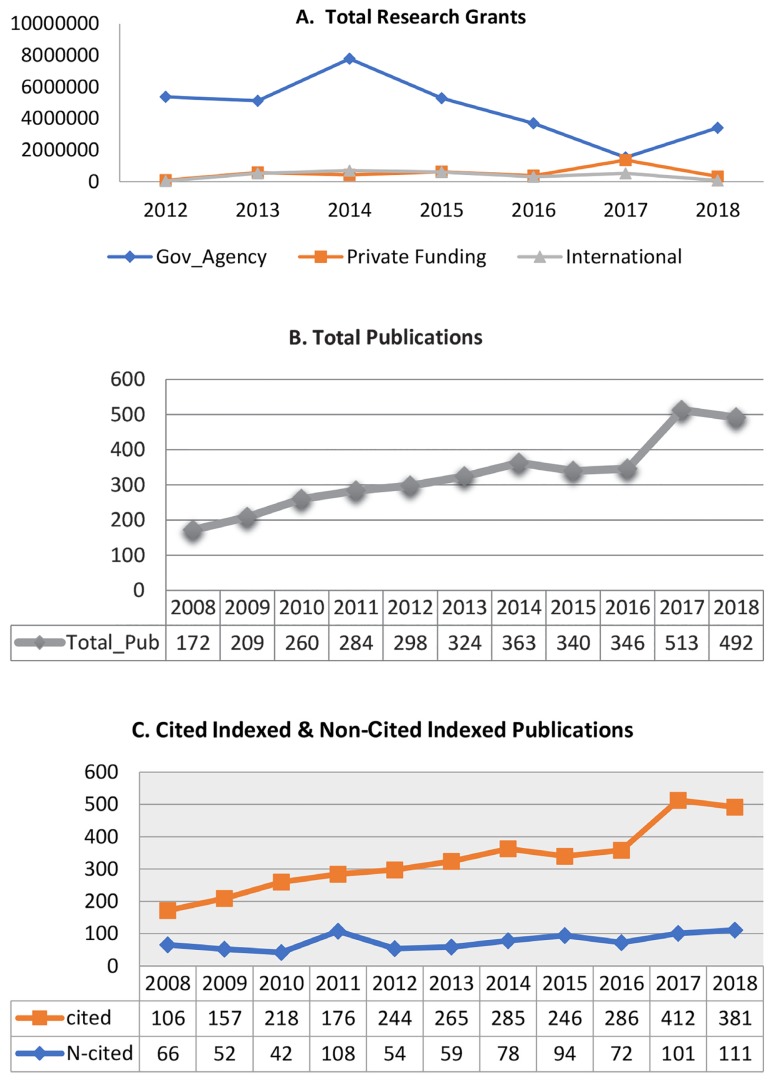
The cumulative performance of research grants (A) and publications (B, C) The six Deputy Deans (Research)—Professor Rashid Abdul Rahman, Professor Nor Hayati Othman, Professor Mustafa Musa, Professor Ahmad Sukari Halim, Professor Nik Soriani Yaacob and Professor Rosline Hassan. For administrative and data management purposes, research in the School of Medical Sciences is being conducted under various clusters, with the supportive research facilities and services (for more details, visit www.medic.usm.my/research/).

In 2008, five research universities were identified by the Ministry of Higher Education, and the USM is one of those, which was subsequently awarded the Accelerated Programme for Excellence (APEX) status (5). The research output is assessed through the Key Performance Indicators Monitoring System (KPI-MS), with the type of grants and publications acquired from ‘USM KPI-MS’. The key performance indicator (KPI) is an index that evaluates the qualitative and quantitative performance of an organisation or institution. The benchmark of the performance indicators for the universities in Malaysia is determined by the Ministry of Education using the Malaysian Research Assessment (MyRA) instrument, which is only applicable to research-intensive universities (RU). The instrument comprises nine sections of measurement. The measurements are on the general information, quantity and quality of researchers, quantity and quality of research, the number of postgraduates, quality of postgraduates, innovations and intellectual property, professional services and gifts, networking and linkages and support services. The KPI-MS is used as a tool in collecting, collating, processing, reporting and monitoring of the KPI data for the measurement mentioned above (Figure 1). To date, the majority of clusters have exceeded RM1 million grant acquisition of research grants. Amongst these, two clusters, namely Cancer and Oncology and the Genetics and Genomics clusters, seem to demonstrate the highest improvement over the monitoring period.

In 2013, USM laid down the APEX phase 2 agenda comprising seven pillars of strength to support the mission and vision of the second phase of the APEX journey. The planning for the school’s research agenda comprises conducting and promoting transdisciplinary research in areas of medical and health sciences, establishing linkages, positioning and enhancing prominence through collaborative research with regional and international research centres; providing medical and disease-related data for national means and improving research clusters towards the development of centre of excellence (COE). Leading strategies involve enhancing cutting-edge research amongst academic staff (strategy 1); intensifying collaborative research with research centres (strategy 2) and enhancing research clusters towards the development of COEs (strategy 3).

## 40 Years of Community and Industry Networking

The school’s section of Community and Industry Networking or Bahagian Jaringan Industri dan Masyarakat (BJIM) was created in 2010 with Associate Professor Col. (B) Dr Wan Pauzi Wan Ibrahim as the first Deputy Dean of BJIM. In 2013, the portfolio became Student Affairs and Network or Hal Ehwal Pelajar dan Jaringan, with close liaisons with USM Deputy Vice-Chancellor Student Affairs and Alumni and the Deputy Vice-Chancellor of the Industry and Community Network.

Corroborating the USM’s second APEX phase planning, ‘to have impactful contributions beyond knowledge discovery and enhancement, in the nurturing and fostering of humanity in a holistic fashion, transcending the physical and the mind, and able to mould ethical citizens towards the transformation and strengthening of civilisation’, the school continues to enhance the existing community engagement programme to a broader network, both at the national and international levels. The strength of the staff and students of the School of Medical Sciences should be completely utilised. Several engagements by staff and departments at the school with various communities have been truly impactful. One such effort was the involvement of the Department of Emergency Medicine in their disaster preparedness during the 2014 Kelantan major flood disaster. The majority of the efforts grew from a strong team of ever-ready volunteers amongst students and staff who are tuned to the appreciation of community and industrial engagements. In addition, the database of the memorandum of agreement (MOA)/understanding (MOU) continues to grow for the school with various agencies, along with the facilitation for medical students’ elective training (many from abroad), as well as industrial training placements within the school clinical and laboratory-based departments in close collaboration with the dedicated teaching, Hospital USM.

## Conclusion

In the last four decades, a wealth of returns for USM, as well as the nation, has been delivered, through thick and thin, with several noteworthy milestones; these achievements were successfully attained despite the pressing challenges and, often, in the setting of scarce resources. Admittedly, without the stewardship of co-founders, past and current leaders, with nurturing of the future emerging leaders for all staff, it would have been tough for this medical school to uphold its vision and mission. Hence, consistent with USM’s core principles (6), the school continually strive to train globally competitive and compassionate medical graduates, medical specialists and scientists, aptly skilled to serve the nation’s needs and global markets. In research and innovation, the school enriched the university medical research ecosystem that hinges on the national development, health and well-being of the community and research leaders for global prominence. As we mature further in years to come, guided by the Malaysian Higher Education Blueprint (2015–2025) enablers and core areas (7), our pursuits shall remain steadfast and align with our core values, respectful integrity and vigilant to eco-sustainable ethos as led by our beloved university, USM ‘We-Lead’ motto.

## Figures and Tables

**Table 1:** Key milestones of the USM School of Medical Sciences since 1979

Year	Milestone
1979	School of Medical Sciences was established in the USM Main Campus, Pulau Pinang
1981	Intake of the first batch of medical students in Pulau Pinang
1983	DYMM Sultan of Kelantan officially opened Hospital USM
1984	The first batch of medical students began their clinical training in Hospital USM
1986	The first batch of medical students graduated/The first MSc student was enrolled
1988	The beginning of Master of Medicine programmes
1989	The first PhD student was enrolled
	
1990	The historic move from USM Main Campus, Pulau Pinang to the USM Health Campus, Kelantan
1992	The first batch of Master of Medicine graduated
1998	Establishment of the School of Dental Sciences (PPSG)/The first batch of MSc in Sports Sciences was enrolled
1999	Establishment School of Health Sciences (PPSK)
2001	Establishment of Clinical Skill Unit/Establishment of Human Genome Centre
2002	School of Dental Sciences officially opened by the Minister of Education
2003	The accreditation of the School of Medical Sciences by the National Accreditation Board/Establishment of Advanced Institute of Medicine and Dentistry (AMDI) in Bertam, Kepala Batas, Pulau Pinang
2003	Establishment of the Institute for Research in Molecular Medicine (INFORMM)
2003	Commencement of Master of Medical Biostatistics, Medical Education and Clinical Anatomy
2004	Clinical Skill Centre officiated by the Minister of Higher Education
2005	USM received the Research University (RU) status
2008	USM received the APEX status
2009	Establishment of an offshore medical programme with the KLE Society, Belgaum, India. USM-KLE IMP (International Medical Programme)
2010	USM-KLE IMP takes in the first batch of medical students
2012	Establishment of a learning centre including 108-bed exam ward, BPSP [Blok Pembelajaran dan Sumber Pelajar]/Commencement of Integrated Neuroscience Programme (INP)
2014	The implementation of the new Doctor of Medicine (MD) medical curriculum (credit unit system)/PPSP Wall of Honour
2015	Graduation of the first batch of USM-KLE IMP students
2018	The implementation of the Shadow House Officer Training Schedule (SHOTS) under the new MD medical curriculum
